# Modifications to Electronic Nicotine Delivery Systems: Content Analysis of YouTube Videos

**DOI:** 10.2196/17104

**Published:** 2020-06-02

**Authors:** Zachary B Massey, Yachao Li, Jessica Holli, Victoria Churchill, Bo Yang, Katherine Henderson, David L Ashley, Jidong Huang, Lucy Popova

**Affiliations:** 1 School of Public Health Georgia State University Atlanta, GA United States; 2 Department of Communication Studies and Department of Public Health The College of New Jersey Ewing, NJ United States; 3 Research and Training Institute John Snow Incorporated Boston, MA United States; 4 Department of Communication University of Arizona Tuscon, AZ United States

**Keywords:** ENDS modifications, YouTube, coils, e-liquid, vaping

## Abstract

**Background:**

As user modification can alter the addictiveness and toxicity of electronic nicotine delivery systems (ENDS), more research is needed to understand the types, motivations, risks, and information sources that lead to these product alterations. YouTube has been identified as a major platform where ENDS users obtain and share information about ENDS products and modifications. However, a comprehensive study of ENDS modification videos on YouTube is lacking.

**Objective:**

This study aimed to analyze the content of YouTube videos depicting modifications of ENDS.

**Methods:**

YouTube was searched in March 2019 to identify videos depicting ENDS modifications. Search terms were derived from interviews with ENDS users and current literature. We used 28 search phrases that combined the words vape and vaping with modification-related key terms (eg, custom build, modification, and dripping). The final sample included 168 videos.

**Results:**

Videos were 1 to 108 min long (median 9.55). Presenters were largely male (117/168, 69.6%), white (94/168, 56.0%), and older than 25 years (94/168, 56.0%). Most videos gave *how to* instructions (148/168, 88.1%), but few offered warnings (30/168, 17.9%) or mentioned commercial alternatives to modifications they presented (16/168, 9.5%). The ENDS devices most often featured were drippers (63/168, 37.5%) and refillable tanks (37/168, 22.0%). The most often modified ENDS components were coils (82/168, 48.8%) and e-liquids (34/168, 20.2%), which included adding other substances, such as cannabis, to the e-liquids (6/168, 3.6%). Most videos portrayed ENDS modifications positively (106/168, 63.1% positive; 60/168, 35.7% neutral; and 2/168, 1.2% negative) and were either neutral or positive in their overall portrayal of ENDS devices (78/168, 46.4% positive; 89/168, 53.0% neutral; and 1/168, 0.6% negative).

**Conclusions:**

This study identified several concerning trends in popular YouTube videos on ENDS modifications, including lack of warnings, the addition of marijuana derivatives to e-liquids, and the positive portrayal of ENDS devices and modifications. By identifying the types of modifications (coil and e-liquid being the most prevalent), this study sets an agenda for research on the effects of modifications.

## Introduction

### Background

Since the mid-2000s, electronic nicotine delivery systems (ENDS) have become an increasingly popular method for inhaling nicotine from tobacco in the United States [1]. As a category, ENDS include a wide variety of products consisting of a battery, a heater (coil), a mouthpiece, and a chamber containing e-liquid (a solution of propylene glycol [PG] or vegetable glycerine [VG] and other chemicals, usually nicotine and flavors). The heater vaporizes the e-liquid, producing an aerosol that is inhaled. ENDS can also be called vapes, e-hookahs, and e-cigarettes. Since June 2019, an outbreak of lung disease linked to vaping and the use of ENDS has sickened 2506 and killed 54 people [2]. Although this outbreak is ongoing, the US Centers for Disease Control and Prevention urges ENDS users not to modify the products or add substances not intended by the manufacturer, highlighting the potential role ENDS modifications might play in this outbreak [3]. For this study, we define *modification* as both product misuse and tampering unintended by the manufacturers as well as alteration, customization, adjustment, and user choice of e-liquid or accessories made within manufacturer specifications. Examples of ENDS modifications include coil replacement, mixing of e-liquids, or increasing battery voltage. Although some users view the modifiability of ENDS devices as a positive attribute [4,5], research has found that modifications can have harmful effects, such as creating higher levels of toxic emissions when users increase power to the coil [6-8]. Given the popularity of ENDS and health risks from modification, research is needed to understand the primary ways ENDS are modified by users.

To date, little research has described the methods of ENDS modifications. YouTube is the most popular video-sharing site in the world [9], and there are a large number of ENDS videos on YouTube [10], including videos about ENDS modifications [11]. YouTube has been identified as a major platform where ENDS users obtain and share information about ENDS products and modifications [12-14]. However, a comprehensive study of ENDS modification videos on YouTube is lacking. One recent study examined YouTube videos depicting orthodox (intended by manufacturer) and unorthodox (unintended by the manufacturer) ENDS modifications [11]. Results showed that videos depicting unorthodox use were three times more prevalent than videos depicting orthodox use, although the analysis only focused on open devices and e-liquids and not on closed devices [11]. Furthermore, the sample was collected in 2016 and may not account for recent trends in ENDS modification. A more in-depth study is needed to update and assess the breadth of ENDS modifications on YouTube.

### Objectives

In this paper, we examined YouTube videos posted between 2013 and 2019 showing modifications of ENDS devices pertaining to hardware (eg, coil, battery, pods, and other modifications) and e-liquids. Our objective was to understand the video features (including presenter characteristics, if applicable) as well as the types, motivations, communication of risks, and information sources of ENDS modifications. Although other studies have examined the social acceptability of ENDS on YouTube [15], unorthodox use of ENDS on YouTube [11], and promotion of vape tricks on YouTube [16], this study, to the best of our knowledge, is the first to analyze modifications to the full spectrum of ENDS devices on YouTube.

## Methods

### Identifying Electronic Nicotine Delivery Systems Modification Videos

YouTube was searched on March 15, 2019, to identify videos depicting ENDS modifications. An account was created on an incognito (private) browser to ensure browsing history did not influence the results. Search terms were derived from interviews with ENDS users [14] and the current literature [10] indicating that “vape” and “vaping” are common and inclusive terms used by ENDS enthusiasts to describe a number of different ENDS devices. Search phrases included the following: “vape DIY (ie, do it yourself),” “vape build DIY,” “vape custom build,” “vape modification,” “vape rebuild DIY,” “vape coil rebuild,” “vape coil DIY,” “vape ohm rebuild,” “vape ohm DIY,” “vape e-juice DIY,” “vape e-juice custom build,” “vape voltage DIY,” “vape voltage custom build,” “vape RBA (ie, rebuildable atomizer) custom build,” “vape RBA DIY,” “vape dripping custom build,” “vape dripping DIY,” “vape refill custom build,” “vape refill DIY,” “vaping custom build,” “vaping DIY,” “vaping modif* custom build,” “vaping rebuild DIY,” “mods rebuild DIY,” “vape chang* DIY,” “vape chang* custom build,” “vape wattage DIY,” and “vape wattage custom build.” Search terms were entered into the YouTube search engine, and the top 10 results for each phrase were included (N=280). After removing duplicates, non-English videos, and those not presenting ENDS modifications, 207 videos remained. Each video was reviewed by trained coders to ensure that the content included ENDS modifications. This verification process excluded 39 videos, resulting in a final sample of 168 videos. This study was approved by the Georgia State University Institutional Review Board (H19055).

### Coding Scheme

We developed a codebook based on interviews with ENDS users [14], previous literature [10,15], and expert consensus. By expert consensus, we refer to review by our interdisciplinary team of collaborators with substantial expertise in ENDS use behavior and ENDS product research. We collected information on the attributes of the videos (eg, length, number of views, number of likes and dislikes, and date of posting). We coded the video source as individual, retailer, manufacturer, or group or organization. We noted whether the videos included links to vendors, whether the videos were followed by other modification videos via YouTube’s auto-play feature (ie, showing content based on user search history), gave *how to* instructions, included a warning (a warning statement about the health risks of smoking or a formal, legal, or conflict of interest disclaimer), or offered information about commercially available alternatives to modifications. If a person appeared in the video, we recorded if it was a single presenter or multiple presenters and their sex (male or female), race (white, black, other, or cannot be determined), and estimated age range (youth: younger than 18 years, young adult: approximately 18-25 years, or adult: older than 25 years). We classified the device(s) featured in the video as dripper, refillable tank, closed pod, refillable pod, home-built refillable tank, cig-a-like, squonk, 2-pod systems, and stem/salt hybrid [17]. Definitions for ENDS devices are provided in Table 1. If multiple devices were shown in a single video, all devices, regardless of their prominence, were included in the coding.

**Table 1:** Content codes for electronic nicotine delivery systems devices (N=168) and the percentage and number of videos in the final sample that featured each device type.

Device type	Definition	Value, n (%)
Dripper	These devices do not include a tank but do include a tip, coil, wicks, battery housing, battery, and fire button. There is no e-liquid reservoir—e-liquid is squirted directly on to the coil housing or *deck* and heated up as soon as the fire button is pressed. They are intended to be taken apart and put back together, which makes them an open system. Some or most of the device has been hand-built, with some parts being retrofitted from existing commercially produced devices.	63 (37.5)
Refillable tank	A commercially produced refillable tank system, which features, at minimum, a tip, a glass tank, a coil, a battery unit, and a fire button. The tip, tank, coil, and batteries can be removed, and the tip, tank, coil, and battery unit can be easily separated from each other and reconnected, which makes it an open device. For commercially produced devices, the battery unit is shaped either like a rectangular box (box mod) or like a cylinder (vape pen).	37 (22.0)
Closed pod	A commercially produced closed pod system with a single pod that cannot be refilled, such as a Juul. These are typically small, thin, rectangular boxes about the shape of a 4-inch long USB drive. However, some may also be parallelogram, square, or teardrop shaped.	7 (4.2)
Refillable pod	A commercially produced closed pod system with a single pod that can be refilled. These are similar to closed pod systems, except that the pods include a small port that allows the e-liquid reservoir to be refilled. Refillable pod devices with only 1 pod usually use salt nicotine (higher concentration and lower wattage).	5 (3.0)
Home-built refillable tank	A home-built device featuring a refillable tank that is not commercially produced. They are intended to be taken apart and put back together, which makes them an open system. Some or most of the device has been hand-built, with some parts being retrofitted from existing commercially produced devices. Home-built parts may include a tip, battery housing, and fire button. Retrofitted parts may include a tip, tank, coil, electronics, and batteries.	4 (2.4)
Cig-a-like	A commercially produced device designed to look like a combustible cigarette in size, shape, and color (sometimes including a brown/tan filter tip). However, it is entirely electronic and includes a battery, a small e-liquid reservoir, and an atomizer. Some are not rechargeable, nor can they be taken apart but are intended to be disposed of once the e-liquid has been used up. Others are rechargeable and have replaceable pods.	4 (2.4)
Squonk	A squonker, also known as squonk mod, is a type of dripper. Like drippers, they include a tip, coil, wicks, battery housing, battery, and fire button. There is, however, a small e-liquid reservoir made out of a pliable material such as silicone, which can be refilled. The e-liquid reservoir is accessible through a hole or window in the device, which allows the reservoir to be compressed. Compressing the reservoir squirts e-liquid directly on the coil, which heats it up as soon as the fire button is pressed. Squonks are only commercially produced and typically look like small box mods without the tank on top of the mod, but with the e-liquid reservoir visible and accessible through a small window on the side.	4 (2.4)
2 pod systems	Devices that include 2 pods of the same type and 2 receptors that can be swapped. This allows the device to provide vapor with 2 distinct flavors as well as vapor that blends the 2 flavors.	0 (0.0)
Stem/salt hybrid	A hybrid that includes 2 refillable pods each with its own receptor on the device. One is intended for salt nicotine e-liquid (higher concentration/lower wattage) and the other is for *stem* nicotine, that is, typical e-liquid (lower concentration of nicotine, if at all/higher wattage). Each pod can only fit into 1 receptor on the device, and they cannot be swapped with the other receptor.	0 (0.0)
Multiple devices	Several devices are featured.	7 (4.2)
Other	Device featured is another product not listed or unsure of the product type.	15 (8.9)

We developed a coding scheme to capture ENDS modifications to the coil, e-liquid, battery, pods, and other modifications. For each type of modification, we developed codes for common reasons for this modification, including an *other* option with text descriptions. Each modification was coded as 1 (present) or 0 (absent). The detailed codes with descriptions for modifications and reasons are presented in Table 2.

Finally, we coded the video’s tone toward the featured ENDS modification and ENDS devices in general as positive, neutral, or negative.

**Table 2:** Codes and definitions of modifications and reasons for modifications (N=168).

Code		Definition	Value^a^, n (%)
**Coil modification**			
	Coil build	Building the coil	60 (35.7)
	Replacing coil	Replacing existing coil with a different coil from the manufacturer	9 (5.4)
	Coil voltage	Altering the voltage of the existing coil through controls built into the device by the manufacturer	8 (4.8)
	Number of coils	Altering the number of coils in the device	1 (0.6)
	Coil gauge	Explicitly replacing an existing coil with a coil that is a different gauge wire	3 (1.8)
	Pod coil	Cleaning the coils built into the pods/closed systems	2 (1.2)
	Other coil modification	Other type of coil modification	35 (20.8)
**Reasons for coil modification**			
	Increased voltage	To increase the voltage of the heating coil	9 (5.4)
	Other	Other reasons for coil modification	31 (18.5)
**E-liquid modification**			
	E-liquid mixing	Mixing e-liquid includes making your own e-liquid from standard components (any of PG^b^, VG^c^, nicotine, flavors, etc) or altering bought e-liquid with any of these components (adding flavors and changing PG/VG ratio)	20 (11.9)
	E-liquid concentration	Replacing e-liquids with different levels of nicotine concentrations	3 (1.8)
	Adding substances	Adding other substance (eg, cannabis oil) or replacing e-liquids with other substances	6 (3.6)
	Other e-liquid modification	Other type of e-liquid modification	13 (7.7)
**Reasons for e-liquid modification**			
	Flavor	To enhance flavors in e-liquid	16 (9.5)
	Cost	To save money/reduce the cost of manufactured liquid	10 (6.0)
	Other	Other reasons for e-liquid modification	15 (8.9)
**Battery modification**			
	Replace battery	Replacing a battery with a battery with different properties	0 (0.0)
	Battery configuration	Changing battery configuration, either in series or in parallel	0 (0.0)
	Additional battery	Attaching additional batteries	0 (0.0)
	Other battery modification	Other modification to battery	13 (7.7)
**Reasons for battery modification**			
	Increased power	To increase the power of the existing tank device	0 (0.0)
	Other	Other reasons for battery modification	4 (2.4)
**Pod modification**			
	Pod refilling	Refilling pods with e-liquids	9 (5.4)
**Reasons for pod modification**			
	Extend life	To extend the life of a pod/cartridge	4 (2.4)
	Other	Other reasons of modifications to closed pod	7 (4.2)
**Other modification**			
	Modifying controls	Modifying software regulating controls	2 (1.2)
	Improved features	Replacing existing device with another device with improved features	1 (0.6)
	Additional features	Replacing existing device with another device with additional features	3 (1.8)
	Other	Other types of modification that are not listed above	22 (13.1)

^a^The number of videos and the percentage in the final sample with each code present.

^b^PG: propylene glycol.

^c^VG: vegetable glycerin.

### Video Coding

The coding team consisted of 5 members, who received a minimum of four 2-hour training sessions. The coders coded 5 to 10 videos for each training session, and discrepancies were discussed and resolved. Training videos were not included in the final data analysis because the code book was further revised during the training. To establish intercoder reliability, approximately 15% of videos (25/168, 14.9%) were randomly selected from the final sample. Due to the large amount of time spent to code each video, sequential-overlapping coding was used for reliability testing [18]. Specifically, each of the 25 videos was coded by 2 coders, and each coder coded 10 videos in total. Using Randolph’s free-marginal kappa [19,20], the intercoder reliability of each variable was high, ranging from 0.74 to 1.0. The remaining videos were then divided and independently coded by 4 coders.

### Data Analysis

Data were analyzed using IBM SPSS 25. Descriptive statistics were performed to assess the frequency of each coding variable.

## Results

### Characteristics of Videos, Presenters, and Device Types

Videos (N=168) ranged from 1 to 108 min in length (median 9.55) and accounted for a total of 112,043,718 views (median 168,234) as of March 15, 2019. Videos had more *likes* than *dislikes* (12 to 1 ratio). Examining videos by year showed an increase from 2013 (2/168, 1.2%) to 2014 (19/168, 11.3%), to 2015 (27/168, 16.1%), to 2016 (34/168, 20.2%), peaking in 2017 (51/168, 30.4%), and decreasing from 2018 (29/168, 17.3%) to 2019 (6/168, 3.6%). The most common source for videos were individuals (135/168, 80.4%), followed by retailers (18/168, 10.7%), manufacturers (8/168, 4.8%), and groups/organizations (7/168, 4.2%). A majority of videos (120/168, 71.4%) provided a link to a vendor’s site, and most videos (136/168, 81.0%) were followed by another modification video via auto-play. Most videos gave *how to* instructions (148/168, 88.1%); few videos offered warnings (30/168, 17.9%) or mentioned commercially available alternatives (16/168, 9.5%).

Most videos showed 1 or more persons (125/168, 74.5%), and presenters were largely male (117/168, 69.6%), white (94/168, 56.0%), and appeared to be adults older than 25 years (94/168, 56.0%). Modifications were most frequently performed on drippers (63/168, 37.5%) and refillable tanks (37/168, 22.0%; Table 3).

**Table 3:** Modification by device type.

Device type	Coil^a^, n	E-liquid^a^, n	Battery^a^, n	Pods^a^, n	Other^a^, n
Dripper	38	7	6	0	3
Refillable tank	28	3	2	0	3
Home-built refillable tank	0	0	2	0	1
Closed pod	0	4	0	6	0
Refillable pod	0	2	0	1	0
Cig-a-like	0	4	0	2	0
Squonk	2	0	0	0	3
Stem/salt hybrid	0	0	0	0	0
Two-pod system	0	0	0	0	0
Multiple devices	3	1	1	0	3
Other	5	1	2	0	9
Total	76	22	13	9	22

^a^Each cell shows the number of videos with each modification. If a video showed a specific modification, the video was coded as 1. Videos coded as 1 include single modifications (eg, building a coil from scratch) or multiple modifications (eg, removing coil, cleaning coil, and rewrapping coil).

### Coil Modifications

Modifications to the coil were the most frequently portrayed; building coils from scratch was featured in 35.7% (60/168) of the videos. Coil replacement with a manufactured alternative was depicted in 5.4% (9/168) of videos, and 4.8% (8/168) of the videos showed how to alter coil voltage through controls in the device. Less than 0.6% (1/168) of the videos gave instructions on how to alter the number of coils in the device, and 1.8% (3/168) showed how to replace coils with a wire of different gauge. Only 1.2% (2/168) of the videos showed how to clean the coils in a nonmodifiable pod.

Reasons for modifying in many of the coil videos (31/168, 18.5%) were not in the prespecified list. Among these reasons, building coils from scratch (17/168, 10.1%) and enhancing flavor (6/168, 3.6%) were the most commonly described. Building coils from scratch included home builds (necessitating a new coil) and adding coils in store-bought devices. According to the presenters, enhancing flavor can be achieved by changing the wire gauge (eg, 28 vs 24 gauge), wire composition (eg, nickel vs stainless steel), or number of wire wraps. Increasing voltage was also listed as a reason for modifying coils (9/168, 5.4%).

The most viewed coil modification video (3,017,588 views) shows a presenter instructing the audience on how to build a sleeper coil (ie, double-wrapped coil made from a single wire). The tutorial includes instructions on how to wrap the coil and attach it to the battery posts. The *how to* portion is book-ended by discussion and demonstration of the modification. Before the tutorial, the presenter describes how the sleeper coil heats e-liquid efficiently, producing a greater cloud and better flavor. After the tutorial, the presenter vapes to demonstrate the size of his aerosol cloud, presumably the result of the sleeper coil.

### Modifications of E-Liquids

Modifications of e-liquids were depicted in 20.2% (34/168) of videos. Many of the e-liquid modification videos showed mixing (20/168, 11.9%; eg, mixing store-bought glycol). Separately, 1.8% (3/168) of videos included mixing liquids to increase nicotine levels. Adding other substances (eg, cannabis oil) was found in 3.6% (6/168) of videos.

Enhancing flavor (16/168, 9.5%) and saving money (10/168, 6.0%) were reasons given for e-liquid modifications, as were *other* reasons (15/168, 8.9%). Descriptions of other reasons included creating better clouds, making nicotine-free liquid, and creating marijuana e-liquid. Overall, e-liquid modifications centered on allowing users to control the flavor, price, and composition of e-liquids.

The most viewed e-liquid mixing video (1,769,171 views) shows how to make e-liquids with store-bought materials. The presenter’s face is not shown, although the video is narrated. The presenter describes 4 components necessary to make e-liquid: VG, PG, premixed liquid nicotine (75% VG and 25% PG), and store-bought flavoring (eg, strawberry and banana). Before mixing the components, the presenter uses a web-based e-liquid calculator. The calculator uses batch size, nicotine strength (mg/ml), and VG/PG ratio to calculate the mixing recipe. However, the calculator does not account for the addition of flavor. Therefore, the presenter explains the conversion necessary to add these liquids. At the completion of the conversion, the presenter combines the liquids in a cylinder and then attaches a clamp to a power drill, wraps the clamp fingers around the cylinder, and engages the drill, spinning the cylinder and mixing the liquids within. The liquid is then *steeped* in warm water to allow the ingredients to blend together. The video closes by encouraging viewers to document mixes in a spreadsheet.

The most viewed *adding substances video* (717,781 views) gives a tutorial on how to make *weed e-cig juice*. The presenter bakes marijuana on a cooking sheet, breaks it apart by hand, and mixes it with VG. After 3 months of iteratively adding glycerin and remixing the solution, the presenter strains marijuana-infused glycerin through a mesh and then injects the liquid into an ENDS tank. The video closes with the presenter blowing vapor clouds over an advertisement for marijuana accessories. Among the *adding substances* videos, most (5 of 6) focused on adding marijuana derivatives to e-liquids.

### Battery Modifications

No videos showed modifications to increase battery power, change configurations, or add additional batteries. The only videos coded for battery modifications were those in the *other battery modification* category (13/168, 7.7%), which showed variations of adding batteries to home-built devices. Examining descriptions for the *other* category showed that all entries were home builds, either adapting household items (eg, a flashlight) or using commercially made parts. Adapting household items to create a vape involved a premade battery, whereas building from scratch sometimes required wiring batteries. This distinction could indicate differences in battery modification hazards.

The most viewed (1,645,630 views) battery modification video transforms a flashlight into an ENDS device. The presenter does not address the audience, but text is displayed to highlight important steps. In the video, the presenter disassembles a flashlight and uses the parts as well as household materials to create a small vape. The body of the flashlight is retrofitted to hold a battery and the head with an atomizer (ie, a coil and wick attached to battery posts housed on a deck). The flashlight button engages the battery, heating the coil and producing a stream of vapor from the atomizer. The battery does not require wiring and appears to be a standard battery consistent with those used in any flashlight.

Other battery videos, however, depict more advanced constructions. One example (763,291 views) shows how to build a *box mod*, which is a highly modifiable ENDS device characterized by a removable atomizer, open tank, and accessible battery housing. The presenter walks the audience through the build, which includes wiring of 2 lithium polymer batteries (ie, LiPo) into the device. LiPos are lightweight batteries that can explode if they are overcharged. Such explosions have been linked to injury of ENDS users [21]. The presenter warns viewers not to let battery wires touch during construction. However, the breadth of the hazard is not disclosed, and the video contains no warnings about the danger of explosions.

### Modifications of Pods and Cartridges

A pod is a cartridge containing e-liquids to be inserted in an ENDS device. When the liquid is gone, many manufacturers intend that the cartridge is discarded and not reused (although there are some refillable pods on the market). Refilling nonreusable pods with e-liquid was found in 5.4% (9/168) of videos. The primary reasons for pod modifications (7/168, 4.2%) were not in the specified list, and thus, they were coded as other. Descriptions of *other* reasons included customizing the look of devices, increasing vapor production, and enhancing flavor. Extending the life of a pod was listed as a reason for 2.4% (4/168) of videos.

An example of pod modification (182,621 views) depicts a presenter changing the liquid in a nonreusable pod. In the video, the presenter uses scissors to open the cartridge. The presenter pours the liquid into another container, cleans the pod, and then refills it with an e-liquid called *Heisenberg* (fruit-flavored menthol). After the refill, the presenter takes several drags until the tobacco flavor is gone, and only Heisenberg remains. In this case, the pod modification is motivated by the choice of the flavor.

### Other Modifications

Only 1.2% (2/168) of videos showed modifications to the software regulating built-in controls. Replacement of an existing device with another with improved features was found in 0.6% (1/168) of videos, whereas replacing an existing device with another device with additional features was featured in 1.8% (3/168) of videos. Although drippers were the most featured devices (63/168, 37.5%), our analyses did not show any instances of converting nondripping devices to drippers.

Finally, 13.1% (22/168) of videos did not fit within our specified coding categories and were coded as *other* modifications. These videos showed building ENDS as the primary activity. Several videos depicted unusual builds, such as creating a vape from an empty mint box or adapting a soda can into an e-liquid tank. One unique example (66,573 views) showed how to build an ENDS in the dashboard of a car. Other videos showed how to make *drip tips* to replace lost mouthpieces for drippers. In one example (85,006 views), the mouthpiece was made from a copper plumbing fitting. Together, videos coded as *other* showed unique modification behaviors mostly related to building from scratch.

### Tone

Most videos used a positive tone to portray ENDS modifications (106/168, 63.1% positive; 60/168, 35.7% neutral; and 2/168, 1.2% negative) but were either neutral or positive in their portrayal of ENDS devices (78/168, 46.4% positive; 89/168, 53.0% neutral; and 1/168, 0.6% negative).

## Discussion

### Principal Findings

This study provides information about the ways in which users modify ENDS devices on YouTube. Results identified several concerning trends, including lack of warnings, addition of marijuana derivatives to e-liquids, and the positive portrayal of ENDS devices and modifications. Regarding specific modifications, the most often modified components were coils and e-liquids. Modifications to the wick and battery were less often discussed. The sample of modification videos did not depict potentially harmful practices identified in past literature, such as modifying a device for *dripping* or increasing battery power. Together, these results help inform public health researchers by identifying trends in ENDS modifications on YouTube, the most popular video-sharing website in the world [9] and a primary source of information on ENDS devices and modifications [11,15,16].

Our findings identified several concerning trends in modification videos. Specifically, the majority of videos gave *how to* instructions without warning viewers about potential dangers (eg, exploding batteries or lung exposure to harmful constituents). The majority also failed to mention commercially available alternatives, which could be less dangerous than home-grown modifications. ENDS devices and modifications were also depicted in a favorable light, and a majority of videos were followed by another modification video, likely the result of YouTube’s default auto-play feature, which queues similar content based on search history. In a situation where YouTube users search for ENDS modifications, favorable portrayals and lack of warnings, coupled with auto-play features, could potentially create a self-reinforcing loop where users search for modification videos. The dangers of modifications are underemphasized to viewers, and these videos are followed by additional videos portraying ENDS in a favorable light, further underemphasizing the dangers of modification. Repeated, user-driven exposure to videos underemphasizing risks could normalize ENDS modifications, which is of particular concern given YouTube’s primary audience.

YouTube is the most popular video-sharing site in the world [9], and YouTube has been identified as a major platform where ENDS users obtain and share information about ENDS products and modifications [12-14]. Many YouTube users are young, with 81% of those aged 15 to 25 years in the United States using YouTube [22]. This demographic is often targeted by tobacco companies [23,24] and is a group that often experiments with cigarettes and ENDS [25]. Positive portrayals of modifications without safety warnings, which are algorithmically linked and shown in sequence to impressionable audiences, bear serious consideration from tobacco control experts. Future research may explore how YouTube modification videos could potentially detract from nicotine cessation campaigns aimed at youth and young adults.

Altering the coil was the most prevalent type of modification in our study. Little research has investigated the effects of exposure to the components of coils on user health. One notable study tested e-liquids for neurotoxicants before and after contact with ENDS devices [26]. Results showed higher levels of toxic metals in the aerosol, suggesting that the contamination resulted from the contact of e-liquids with the metals in ENDS devices, including coils. As coil modifications were most prevalent in our analysis, we suggest future research investigating possible health effects of coil modifications.

E-liquid was the second most often modified characteristic in our sample. E-liquid modifications included mixing from scratch and adding other substances. In terms of mixing, most videos cited a desire to control the flavor as a reason for the modification. This finding is informative given the recent interest among policy makers to ban flavored e-liquids from the market [27]. Regulatory policy should consider how regulating ENDS flavors might influence the likelihood that these actions will impact the frequency at which consumers mix their own flavored e-liquids.

Marijuana derivatives were used in most videos depicting substances added to e-liquids. This finding is significant given the recent outbreak of vaping-related lung disease [28]. Early investigation revealed that the majority of patients had a history of vaping tetrahydrocannabinol (THC) products, suggesting that the outbreak may be related to the source of THC or vaping THC in a device intended to be used with nicotine [29]. Given the deadly nature of the new vaping lung disease, this particular modification may demand greater scrutiny from public health experts.

Tampering with devices to expose heating coils for dripping has been cited as a modification trend in past research [30]. Yet, none of the videos in our sample showed how to adapt a nondripping device into a dripper. This finding resonates with recent interviews of ENDS enthusiasts, indicating that dripping modifications may be less frequent due to increased availability of commercially produced drippers and other devices that were previously only available via users’ modifications [14]. However, it should be noted that drippers were the most featured devices in YouTube videos. Thus, although dripping conversions were absent in our sample, modifications to drippers were frequent.

Although exploding batteries are a risk [6], we found only a few videos on battery modifications. They were generally associated with building ENDS mods from scratch and showed some dangerous practices (eg, wiring LiPo batteries) that have not been widely discussed previously.

### Limitations

As this sample was collected in early 2019, it is unclear if the decline in videos on ENDS modifications we observed since 2017 will continue. Although this decrease corroborates our findings from interviews with ENDS enthusiasts [14], more research is needed to determine if this decrease continues. As this study was conducted before the first cases of e-cigarette– and vaping-associated lung injury were reported, we do not know what impact this outbreak may have had on ENDS modification activities. Our search keywords were not exhaustive and may not capture all ENDS modification videos on YouTube. Due to the variation of ENDS modification videos, our results are descriptive. More research is needed to isolate and identify causal mechanisms that motivate modification behaviors. This work informs future population-level surveillance research aimed at identifying motivations for modifying ENDS devices.

### Conclusions

Our content analysis of the full spectrum of ENDS devices and modifications in YouTube videos identified several concerning trends, including lack of warning to viewers, addition of marijuana derivatives to e-liquids, and the positive portrayal of ENDS devices and modifications. At the same time, our analysis did not find certain modifications (eg, dripper conversions or battery tampering), which have been identified previously as a public health concern. By identifying the types of modifications (with modifications to coil and e-liquid being the most prevalent), this study provides a foundation for assessing the prevalence of ENDS modifications in the population and informs the agenda for research assessing the health effects resulting from ENDS modifications.

## Abbreviations

DIY: do it yourself
ENDS: electronic nicotine delivery systems
PG: propylene glycol
RBA: rebuildable atomizer
THC: tetrahydrocannabinol
VG: vegetable glycerin

## References

[1] McMillen RC, Gottlieb MA, Shaefer RM, Winickoff JP, Klein JD (2015). Trends in electronic cigarette use among US adults: use is increasing in both smokers and nonsmokers. Nicotine Tob Res.

[2] (2019). Centers for Disease Control and Prevention.

[3] (2020). Centers for Disease Control and Prevention.

[4] Bala MM, Strzeszynski L, Topor-Madry R (2017). Mass media interventions for smoking cessation in adults. Cochrane Database Syst Rev.

[5] Majmundar A, Kirkpatrick M, Cruz TB, Unger JB, Allem JP (2019). Characterising KandyPens-related posts to Instagram: implications for nicotine and cannabis use. Tob Control.

[6] Kosmider L, Sobczak A, Fik M, Knysak J, Zaciera M, Kurek J, Goniewicz ML (2014). Carbonyl compounds in electronic cigarette vapors: effects of nicotine solvent and battery output voltage. Nicotine Tob Res.

[7] Sleiman M, Logue JM, Montesinos VN, Russell ML, Litter MI, Gundel LA, Destaillats H (2016). Emissions from electronic cigarettes: key parameters affecting the release of harmful chemicals. Environ Sci Technol.

[8] Talih S, Balhas Z, Salman R, Karaoghlanian N, Shihadeh A (2016). 'Direct dripping': a high-temperature, high-formaldehyde emission electronic cigarette use method. Nicotine Tob Res.

[9] Alexa.

[10] Huang J, Kornfield R, Emery SL (2016). 100 million views of electronic cigarette YouTube videos and counting: quantification, content evaluation, and engagement levels of videos. J Med Internet Res.

[11] Guy MC, Helt J, Palafox S, Green K, Soule EK, Maloney SF, Eissenberg T, Fagan P (2019). Orthodox and unorthodox uses of electronic cigarettes: a surveillance of YouTube video content. Nicotine Tob Res.

[12] McCausland K, Maycock B, Leaver T, Jancey J (2019). The messages presented in electronic cigarette-related social media promotions and discussion: scoping review. J Med Internet Res.

[13] Collins L, Glasser AM, Abudayyeh H, Pearson JL, Villanti AC (2019). E-cigarette marketing and communication: how e-cigarette companies market e-cigarettes and the public engages with e-cigarette information. Nicotine Tob Res.

[14] Li Y, Fairman RT, Churchill V, Ashley DL, Popova L (2020). Users' modifications to electronic nicotine delivery systems (ENDS): interviews with ENDS enthusiasts. Int J Environ Res Public Health.

[15] Luo C, Zheng X, Zeng DD, Leischow S (2014). Portrayal of electronic cigarettes on YouTube. BMC Public Health.

[16] Kong G, LaVallee H, Rams A, Ramamurthi D, Krishnan-Sarin S (2019). Promotion of vape tricks on YouTube: content analysis. J Med Internet Res.

[17] Harrell PT, Eissenberg T (2018). Automated dripping devices for vapers: RDTAs, bottomfeeders, squonk mods and dripboxes. Tob Control.

[18] Neuendorf KA (2016). The Content Analysis Guidebook.

[19] Randolph JJ (2005). Free-Marginal Multirater Kappa (multirater K[free]): An Alternative to Fleiss' Fixed-Marginal Multirater Kappa. Proceedings of the Joensuu Learning and Instruction Symposium.

[20] Randolph JJ (2008). Justus Randolph.

[21] Nicoll KJ, Rose AM, Khan MA, Quaba O, Lowrie AG (2016). Thigh burns from exploding e-cigarette lithium ion batteries: first case series. Burns.

[22] Clement J (2019). Statista.

[23] Henriksen L, Schleicher NC, Dauphinee AL, Fortmann SP (2012). Targeted advertising, promotion, and price for menthol cigarettes in California high school neighborhoods. Nicotine Tob Res.

[24] Pierce JP, Messer K, James LE, White MM, Kealey S, Vallone DM, Healton CG (2010). Camel No. 9 cigarette-marketing campaign targeted young teenage girls. Pediatrics.

[25] (2015). Centers for Disease Control and Prevention.

[26] Olmedo P, Goessler W, Tanda S, Grau-Perez M, Jarmul S, Aherrera A, Chen R, Hilpert M, Cohen JE, Navas-Acien A, Rule AM (2018). Metal concentrations in e-cigarette liquid and aerosol samples: the contribution of metallic coils. Environ Health Perspect.

[27] (2019). US Food and Drug Administration.

[28] Perrine CG, Pickens CM, Boehmer TK, King BA, Jones CM, de Sisto CL, Duca LM, Lekiachvili A, Kenemer B, Shamout M, Landen MG, Lynfield R, Ghinai I, Heinzerling A, Lewis N, Pray IW, Tanz LJ, Patel A, Briss PA, Lung Injury Response Epidemiology/Surveillance Group (2019). Characteristics of a multistate outbreak of lung injury associated with e-cigarette use, or vaping - United States, 2019. MMWR Morb Mortal Wkly Rep.

[29] Lewis N, McCaffrey K, Sage K, Cheng C, Green J, Goldstein L, Campbell H, Ferrell D, Malan N, la Cross N, Maldonado A, Board A, Hanchey A, Harris D, Callahan S, Aberegg S, Risk I, Willardson S, Carter A, Nakashima A, Duncan J, Burnett C, Atkinson-Dunn R, Dunn A (2019). E-cigarette use, or vaping, practices and characteristics among persons with associated lung injury-Utah, April-October 2019. MMWR Morb Mortal Wkly Rep.

[30] Brown CJ, Cheng JM (2014). Electronic cigarettes: product characterisation and design considerations. Tob Control.

